# One thousand patients with essential thrombocythemia: the Mayo Clinic experience

**DOI:** 10.1038/s41408-023-00972-x

**Published:** 2024-01-18

**Authors:** Naseema Gangat, Omer Karrar, Aref Al-Kali, Kebede H. Begna, Michelle A. Elliott, Alexandra P. Wolanskyj-Spinner, Animesh Pardanani, Curtis A. Hanson, Rhett P. Ketterling, Ayalew Tefferi

**Affiliations:** 1 Division of Hematology, Mayo Clinic, Rochester, MN USA; 2 Division of Hematopathology, Mayo Clinic, Rochester, MN USA; 3 Division of Laboratory Medicine and Cytogenetics, Mayo Clinic, Rochester, MN USA

**Keywords:** Myeloproliferative disease, Myeloproliferative disease

## Abstract

We describe 1000 patients with essential thrombocythemia seen at the Mayo Clinic between 1967 and 2023: median age 58 years (18–90), females 63%, *JAK2*/*CALR*/*MPL*-mutated 62%/27%/3%, triple-negative (TN) 8%, extreme thrombocytosis (ExT; platelets ≥1000 × 10^9^/L) 26%, leukocytosis (leukocyte count >11 × 10^9^/L) 20%, and abnormal karyotype 6%. *JAK2*-mutated patients were older (median 71 years), and *CALR* mutated (52 years), and TN (50 years) younger (*p* < 0.01). Female gender clustered with TN (73%) and *JAK2* (69%) vs. *CALR*/*MPL* (49%/47%) mutations (*p* < 0.01). ExT clustered with *CALR* (type-2 more than type-1) and TN and leukocytosis with *JAK2* mutation (*p* < 0.01). In multivariable analysis, risk factors for overall survival were older age (*p* < 0.01), male gender (HR 1.8), absolute neutrophil count (ANC) ≥ 8 × 10^9^/L (HR 1.6), absolute lymphocyte count (ALC) < 1.7 × 10^9^/L (HR 1.5), hypertension (HR 1.7), and arterial thrombosis history (HR 1.7); for leukemia-free survival, ExT (HR 2.3) and abnormal karyotype (HR 3.1); for myelofibrosis-free survival, ANC ≥ 8 × 10^9^/L (HR 2.3) and *MPL* mutation (HR 3.9); for arterial thrombosis-free survival, age ≥60 years (HR 1.9), male gender (HR 1.6), arterial thrombosis history (HR 1.7), hypertension (HR 1.7), and *JAK2* mutation (HR 1.8); for venous thrombosis-free survival, male gender (HR 1.8) and venous thrombosis history (HR 3.0). Associations between ExT and leukemic transformation and between ANC and fibrotic progression were limited to *JAK2*-mutated cases. Aspirin therapy appeared to mitigate both arterial (HR 0.4) and venous (HR 0.4) thrombosis risk. HR-based risk models delineated patients with median survivals ranging from 10 years to not reached and 20-year leukemia/myelofibrosis incidences from 3%/21% to 12.8%/49%. The current study provides both novel and confirmatory observations of essential thrombocythemia.

## Introduction

Essential thrombocythemia (ET) is one of four *JAK2* mutation-prevalent myeloproliferative neoplasms (MPNs) and is characterized by a mandatory but not specific thrombocytosis (platelet count ≥450 × 109/L) that is proven or presumed to be clonal and not associated with another myeloid neoplasm, such as chronic myeloid leukemia (CML), polycythemia vera (PV), and primary myelofibrosis (PMF) [1, 2]. The latter two share common molecular and morphologic traits with ET, including *JAK2, CALR*, and *MPL* mutations (also known as MPN driver mutations); these mutations are mutually exclusive, for the most part, and their frequencies in ET are ~60% for *JAK2*, 25% for *CALR*, and 3% for *MPL*; of note, these three driver mutations might not be detected in ~10–15% of patients with ET, henceforth referred to as triple-negative ET [1, 3].

Prognosis in ET is generally favorable with consistent risk of thrombohemorrhagic complications and disease progression into myelofibrosis (post-ET MF) or acute myeloid leukemia (AML), also known as “blast phase MPN” [4–6]. Survival in ET approximates that of the general population with median estimated to exceed 30 years in patients younger than 40 years of age [7–9]. The recently introduced “triple A (AAA)” survival model in ET employs age, absolute neutrophil count, and absolute lymphocyte count, in order to risk-stratify patients into high, intermediate-2, intermediate-1, and low-risk groups, with respective median survivals of 8, 13.5, 20.7, and 47 years [10]. In addition, abnormal karyotype [11] and high molecular risk (HMR-ET; *SF3B1*, *TP53*) mutations [12] independently predict inferior survival in ET. Current drug therapy has not been shown to modify the natural history of the disease, and its use is primarily directed at the prevention of thrombosis, guided by thrombosis risk models that are based on thrombosis history, age, and presence of *JAK2* mutation [13, 14].

Over the last half-century, the Mayo Clinic has been a center of excellence for patient care and research in MPN, under the leadership of the late Murray N. Silverstein (1928–1998); part of this decades-long experience has been assembled into previously published large natural history studies, including a 1000-patient report on primary myelofibrosis [15]. The current report includes 1000 patients with ET, seen at the Mayo Clinic between 1967 and 2023, and selected on the basis of full annotation for driver mutations; we describe presenting clinical and laboratory characteristics, frequency and outcome of post-diagnosis events, and detailed global and driver mutation-specified analyses of overall, leukemia-free, and myelofibrosis-free survival, as well as predictors for such events.

## Methods

The current study includes 1000 consecutive patients with ET, who underwent evaluation at the Mayo Clinic between December 1967 and March 2023 and in whom bone marrow biopsies and driver mutation information was available for review. All cases fulfilled the ICC 2022 diagnostic criteria [16] and were fully annotated for driver mutations, while cytogenetic information was available in a subset of patients (*n* = 875). Patients were retrospectively recruited after institutional review board approval was obtained. In order to minimize the inadvertent inclusion of patients with masked PV [17], *JAK2* mutated cases with hemoglobin level >16 g/dL in women and 16.5 g/dL in men were excluded; similarly, cases with anemia defined by sex adjusted hemoglobin level of <11 g/dL in women and <12.5 g/dL in men without an alternative explanation were also excluded, in order to avoid inadvertent inclusion of patients with prefibrotic MF [18]. Thrombosis and survival risk was assessed by the revised IPSET-thrombosis [13] and triple A survival model [10], respectively. Conventional criteria were used for definitions of major arterial and venous thrombotic events, major hemorrhage, fibrotic or leukemic transformation [4, 6, 16]. Therapeutic interventions were dependent on physician discretion and mostly included aspirin therapy in low-risk patients and the addition of cytoreductive therapy, in high-risk patients. Patients were followed until death or last follow-up, as assessed by medical records or through direct contact with patients or their physicians, with follow-up information updated in August 2023.

Comparison between categorical variables was performed by Chi-square test and continuous variables by Wilcoxon/Kruskal–Wallis tests. Cox regression analysis was used to identify risk factors for overall (OS), leukemia-free survival (LFS), myelofibrosis-free (MFFS), and thrombosis-free (TFS). The Kaplan–Meier method was used to construct time-to-event curves, which were compared by the log-rank test. *P* value ≤ 0.05 was considered significant. JMP Pro 17.1.0 software package, SAS Institute, Cary, NC was utilized for all analyses.

## Results

### Presenting characteristics

One thousand patients with ET (median age 58 years, 63% female) were fully annotated for driver mutations which included *JAK2* (62%, *n* = 617), *CALR* (27%, *n* = 269 [type 1/type 1-like *CALR*, *n* = 149; type 2/type-2-like *CALR, n* = 105, *CALR* type indeterminate, *n* = 15]), *MPL* (3%, *n* = 30), or TN (8%, *n* = 84). Information regarding presenting clinical and laboratory features, including treatment, was available in most patients (Table 1). Median age/gender distributions for *JAK2*, type 1/type 1-like *CALR*, type 2/type 2-like *CALR, MPL*-mutated, and TN cases were 71 years/69% females, 53 years/50% females, 51 years/51% females, 66 years/47% females, and 50 years/73% females, respectively (*p* < 0.01/< 0.01). Median values for hemoglobin, platelet, and leukocyte count were 13.9 g/dL, 777 × 10^9^/L (extreme thrombocytosis (ExT); platelets ≥1000 × 10^9^/L in 26%) and 8.5 × 10^9^/L (leukocyte count >11 × 10^9^/L in 20%), respectively. Median/range values for ANC (*n* = 653) were 5.69 (1.54–26.5) (ANC ≥ 8 × 10^9^/L in 17%), and ALC *(n* = 650) were 1.86 (0.38–5.69) (ALC < 1.7 × 10^9^/L in 40%). Palpable splenomegaly was documented in 12% patients, microvascular symptoms in 29% and cardiovascular risk factors in 54%. Cytogenetic studies showed an abnormal karyotype in 6% with incidence rates of 7%, 4%, 3%, and 4%, in *JAK2*/*CALR*/*MPL*-mutated and TN cases, respectively, (*p* = 0.31). Treatment information was available in most patients; aspirin was initiated at the time of diagnosis in 763/908 (81%), cytoreductive therapy in 563/915 (62%), and systemic anticoagulation in 167/859 (19%) of patients.

**Table 1:** Presenting clinical and laboratory characteristics of 1000 patients with essential thrombocythemia (ET), fully annotated for driver mutations, stratified by diver mutation (*JAK2, CALR, MPL, triple negative*).

Variables	All patients *n* = 1000	*JAK2* mutated *n* = 617 (62%)	*CALR* mutated *n* = 269 (27%)	*MPL* mutated *n* = 30 (3%)	Triple negative *n* = 84 (8%)	*P* value *JAK2 vs CALR*	*P* value *JAK2 vs MPL*	*P* value *JAK2* vs triple negative	*P* value *CALR* vs triple negative
Age in years, median (range)	58 (18–90)	71 (18–90)	52 (18–85)	66 (36–89)	50 (19–86)	**<0.0001**	**0.02**	**<0.0001**	0.26
Age ≥60 years, *n* (%)	449 (45)	305 (49)	93 (35)	23 (77)	28 (33)	<**0.0001**	**0.003**	**0.005**	0.83
Female gender, *n* (%)	633 (63)	427 (69)	131 (49)	14 (47)	61 (73)	**<0.0001**	**0.01**	0.52	**<0.0001**
Hemoglobin g/dl, median (range)	13.9 (10–17.2)	14.0 (10–17.2)	13.6 (10.5–16.4)	13.8 (11.1–16.1)	13.3 (11.5–15.8)	**0.002**	0.15	**<0.0001**	**0.02**
Leukocyte count, 10^9^/l, median (range)	8.5 (3.5–23)	8.9 (3.5–23)	8.0 (3.5–18.6)	7.4 (4.3–11.6)	7.8 (3.5–19.6)	**<0.0001**	**0.001**	**0.02**	0.44
Leukocyte count > 11 × 10^9^/l, *n* (%)	197 (20)	147 (24)	33 (12)	1 (3)	16 (19)	<**0.0001**	**0.002**	0.19	0.13
Platelet count, 10^9^/l, median (range)	777 (450–3460)	705 (450–2466)	955 (454–3460)	802 (551–1169)	905 (473–3330)	**<0.0001**	0.55	**<0.0001**	0.94
Platelet count ≥ 1000 × 10^9^/l, *n* (%)	264 (26)	105 (17)	123 (46)	5 (17)	31 (37)	<**0.0001**	0.96	<**0.0001**	0.15
Platelet count ≥ 1500 × 10^9^/l, *n* (%)	48 (5)	16 (3)	22 (8)	0 (0)	10 (12)	**0.0003**	0.21	**0.0004**	0.31
Cardiovascular risk factors, *n* (%)	506/944 (54)	334/588 (57)	114/249 (46)	17/29 (59)	41/78 (53)	**0.004**	0.85	0.48	0.30
Diabetes mellitus	83/942 (9)	49/588 (8)	22/248 (9)	3/29 (10)	9/77 (12)	0.80	0.71	0.35	0.47
Hypertension	405/943(43)	271/588 (46)	89/249 (36)	12/29 (41)	33/77(43)	**0.006**	0.62	0.59	0.26
Smoking	189/940 (20)	124/587 (21)	36/248 (15)	8/29 (28)	21/76 (28)	**0.02**	0.42	0.21	**0.01**
Palpable splenomegaly, *n* (%)	120/990 (12)	77/609 (13)	30/267(11)	2/30 (7)	11/84 (13)	0.55	0.29	0.91	0.65
Abnormal karyotype, *n* (%)	54/875 (6)	40/546 (7)	10/228 (4)	1/29 (3)	3/72 (4)	0.12	0.38	0.29	0.94
Major thrombosis at or prior to diagnosis, *n* (%)	222 (22)	164 (27)	29 (11)	5 (17)	24 (29)	**<0.0001**	0.21	0.63	**0.0001**
Arterial thrombosis^a^	137 (14)	98 (16)	20 (7)	4 (13)	15 (18)	**0.0004**	0.70	0.65	**0.01**
Venous thrombosis^b^	102 (10)	80 (13)	10 (4)	2 (7)	10 (12)	<**0.0001**	0.27	0.78	**0.01**
Major hemorrhage at or prior to diagnosis^c^, *n* (%)	74/983 (8)	47/609 (8)	13/262 (5)	3/30 (10)	11/82 (13)	0.13	0.66	0.10	**0.01**
Microvascular symptoms^d^, *n* (%)	282/958 (29)	180/596 (30)	73/254 (29)	10/29 (34)	19/79 (24)	0.67	0.63	0.25	0.41
Revised IPSET-thrombosis^e^, *n* (%)									
Very low	57 (6)	0 (0)	47 (17)	3 (10)	6 (7)	**–**	**–**	**–**	**0.002**
Low	256 (26)	79 (13)	130 (48)	10 (33)	37 (44)				
Intermediate	191 (19)	94 (15)	65 (24)	12 (40)	20 (24)				
High	496 (50)	444 (72)	27 (10)	5 (17)	21 (25)				
IPSET survival^f^, *n* (%)						<**0.0001**	**0.01**	0.07	**0.05**
Low	371 (37)	187 (30)	146 (54)	5 (17)	33 (39)				
Intermediate	445 (45)	290 (47)	93 (35)	22 (73)	40 (48)				
High	184 (18)	140 (23)	30 (11)	3 (10)	11 (13)				
Treatment instituted at diagnosis, *n* (%)									
Aspirin	736/908 (81)	482/575 (84)	170/231 (74)	25/29 (86)	59/73 (81)	**0.001**	0.72	0.52	0.20
Cytoreductive therapy^g^	563/915 (62)	340/574 (59)	157/238 (66)	20/28 (71)	46/75 (61)	0.07	0.19	0.73	0.67
Systemic anticoagulation	167/859 (19)	119/527 (23)	32/229 (14)	4/28 (14)	12/75 (16)	**0.005**	0.28	0.18	0.47

^a^Major arterial thrombosis includes myocardial infarction, angina, cerebrovascular accidents, transient ischemic attack, peripheral arterial thrombosis, aortic thrombosis, mesenteric artery thrombosis, central retinal thrombosis.

^b^Major venous thrombosis includes deep venous thrombosis, pulmonary embolism, portal/splenic/mesenteric/hepatic vein thrombosis, cerebral sinus thrombosis.

^c^Major hemorrhage includes bleeding events that require red cell transfusion support, resulted in ≥2 g/dl decline in hemoglobin or involved critical organs.

^d^Microvascular symptoms include headaches, paresthesia, erythromelalgia.

^e^International prognostic score for thrombosis in ET (IPSET-thrombosis).

^f^International prognostic score for survival in ET (IPSET-survival).

^g^cytoreductive therapies included hydroxyurea, anagrelide, interferon, busulphan.

Bold value boxes represent variables of significance.

Table 1 lists presenting features of 1000 patients with ET and highlights salient associations of genotype with phenotype. At presentation, *JAK2-*mutated patients compared with *CALR-*mutated counterparts were significantly older (median age; 71 vs 52 years; *p* < 0.01), displayed female preponderance (69% vs 49%; *p* < 0.01), higher incidence of hypertension (46 vs 36%; *p* < 0.01), and smoking (21% vs 15%; *p* = 0.02), higher hemoglobin (median Hb; 14 vs 13.6 g/dl; *p* < 0.01), and leukocyte count (median leukocyte count; 8.9 vs 8 × 10^9^/l; *p* < 0.01), lower platelet count (median platelet count; 705 vs 955 × 10^9^/l; *p* < 0.01) and higher rates of arterial (16% vs 7%; *p* < 0.01) and venous thrombosis history (13% vs 4%; *p* < 0.01). Findings were similar when *JAK2-*mutated cases were compared with those harboring type 1/type 1-like or type 2/type 2-like *CALR* mutations (Supplementary Table 1). Overall, type 1/type 1-like and type 2/type 2-like *CALR-*mutated patients depicted similar phenotype with the exception of higher platelet count in the presence of type 2/type 2-like *CALR* mutation (median; 1044 vs 890 × 10^9^/l; *p* = 0.001) (Supplementary Table 1). Compared with *MPL-*mutated cases, *JAK2*-mutated patients were older (median age; 71 vs 66 years; *p* = 0.02), more likely to be female (69 vs 47%; *p* = 0.01) and displayed higher leukocyte count (median leukocyte count; 8.9 vs 7.4 × 109/l; *p* = 0.001). *JAK2-*mutated and TN patients shared similar gender distribution (predominantly female), with notable differences in age (older age for *JAK2*-mutated), hemoglobin level (higher in *JAK2-*mutated), leukocyte count (higher in *JAK2*-muated) and platelet count (higher in TN). On the other hand, comparison of *CALR*-mutated and TN patients revealed the former to be associated with male gender, higher hemoglobin level, and lower rates of arterial and venous thrombosis and major hemorrhage history (Table 1).

Major thrombosis history at or before diagnosis was present in 222 (22%) of patients, including 137 (14%) arterial, and 102 (10%) venous events. Incidence rates of major arterial/venous thrombosis for *JAK2*, type 1/type 1-like *CALR*, type 2/type 2-like *CALR, MPL*-mutated and TN cases were 16%/13%, 7%/5%, 7%/2%, 13%/7%, and 18%/12%, respectively. Arterial and venous thrombosis rates were significantly lower in *CALR*-mutated cases when compared with *JAK2*, whose thrombosis risk was otherwise similar to those with MPL mutation and TN (*p* < 0.01 and <0.01). Furthermore, advanced age, male gender, and hypertension showed an independent association with arterial thrombosis at or prior to diagnosis (Table 2). 74 of 983 (8%) patients reported major hemorrhage history with respective incidence rates of 8%, 4%, 7%, 10%, and 13%, for *JAK2*, type 1/type 1-like *CALR*, type 2/type 2-like *CALR, MPL*-mutated and TN cases. Notably, *CALR*-mutated patients were less likely to present with major hemorrhage compared to *JAK2, MPL*-mutated, and TN cases (5% vs 10%; *p* = 0.05). On the other hand, female gender was associated with higher rates of hemorrhage (76% vs 24% in female and male patients; *p* = 0.02) (Table 2).

**Table 2:** Univariate and multivariable analysis of associations/risk factors for vascular events (arterial/venous thrombosis and hemorrhage) at or after diagnosis among 1000 patients with essential thrombocythemia (ET), fully annotated for driver mutations.

*Cardiovascular risk factor includes the presence of diabetes mellitus, hypertension, or smoking. Red shaded boxes represent variables of significance. Gray boxes represent variables that were not computed.

### Clinical course

#### Survival

Among 1000 patients with ET, 282 (28%) were followed until death; the median follow-up time for all patients and living patients was 8.5 years (range, 0.01–52.7) and 7.1 years (range, 0.01–52.7), respectively. Causes of death were known in 124 patients and included blastic transformation (*n* = 26), infection (*n* = 26), thrombosis (*n* = 16), solid tumor (*n* = 15), major hemorrhage (*n* = 8), heart failure (*n* = 6), myelofibrosis (*n* = 5), dementia (*n* = 4), renal failure (*n* = 5), hepatic failure (*n* = 3), injury/accident (*n* = 3), graft-versus host disease (*n* = 2) and others (*n* = 8).

**Fig. 1:**
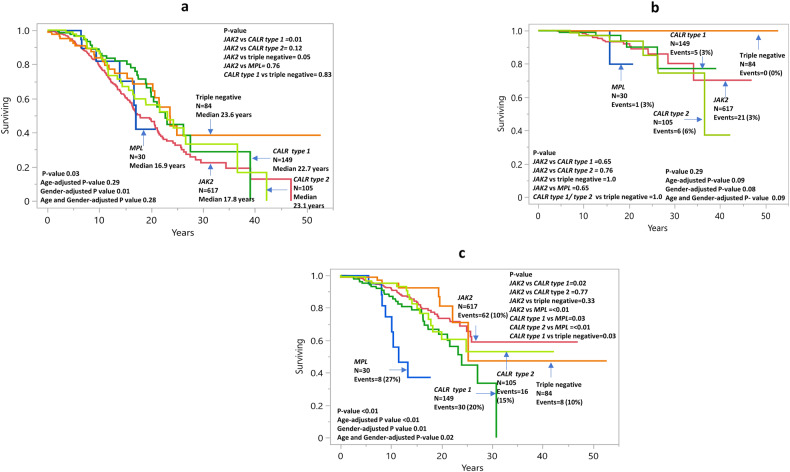
Survival and leukemic/myelofibrotic transformation in essential thrombocythemia. **a** Overall survival in 985 patients with essential thrombocythemia stratified by driver mutation. **b** Leukemia-free survival in 985 patients with essential thrombocythemia stratified by driver mutation. **c** Myelofibrosis-free survival in 985 patients with essential thrombocythemia stratified by driver mutation.

Median survival was 20.6 years with 10-year, 20-year, and 30-year survival rates of 81%, 52%, and 25%, respectively. In univariate analysis, OS appeared significantly better in type 1/type 1-like *CALR*, type 2/type 2-like *CALR*-mutated and TN patients (median 23.1/23.6/22.7 years, respectively) and worse in *MPL* and *JAK2-*mutated cases (median 16.9/17.8 years) (*p* = 0.03) (Fig. 1a). However, the difference in OS was no longer apparent (*p* = 0.39) during multivariable analysis that included age and gender, which were differentially clustered with specific driver mutations (Table 1). Moreover, univariate analysis also identified the following variables as risk factors for OS: age >70 years and 50–70 years, ANC ≥ 8 × 10^9^/L, ALC < 1.7 g/dl, male gender, arterial thrombosis history, hypertension, abnormal karyotype, arterial thrombosis, and major hemorrhage after diagnosis. In multivariable analysis, age >70 years and 50–70 years (*p* < 0.01; HR 22.4/5.1, 95% CI 11.7–43.2/2.8–9.6, respectively), ANC ≥ 8 × 10^9^/L (*p* < 0.01; HR 2.4, 95% CI 1.6–3.4), ALC < 1.7 g/dl (*p* = 0.02; HR 1.5, 95% CI 1.1–2.1), male gender (*p* = 0.01; HR 1.8, 95% CI 1.3–2.7), arterial thrombosis history (*p* = 0.01; HR 1.7, 95% CI 1.1–2.7), and hypertension (*p* = 0.01; HR 1.7, 95% CI 1.1–2.6) were independently predictive of inferior survival (Table 3).

**Table 3:** Univariate and multivariable analysis of risk factors for disease transformation (myelofibrosis and acute leukemia) and overall survival among 1000 patients with essential thrombocythemia (ET), fully annotated for driver mutations.

Follow-up in years, median (range): 8.5 (0.01–52.7)	Overall survival			Leukemia-free survival		Myelofibrosis- free survival	
	Events; *n* (%): 282 (28)			Events; *n* (%): 33 (3)		Events; *n* (%): 126 (13)	
Variables	Univariate *P* value	Age-adjusted *P* value	Multivariable *P* value (HR, 95% CI)	Univariate *P* value	Multivariable *P* value (HR, 95% CI)	Univariate *P* value	Multivariable *P* value (HR, 95% CI)
Age	**<0.0001**			0.14		**0.04**	0.07
Age categories (>70 years, 50–70 years, <50 years)	**<0.0001**		**<0.0001** **>70 vs <50 yrs** **22.1 (11.7–43.2)** **>70 vs 50–70 yrs** **5.1 (2.8–9.6)**	0.33		0.12 >70 vs <50 yrs 0.17 50–70 vs <50 yrs 0.06	0.18
Gender	**0.01 Male**	**0.01 Male**	**0.01 Male** **1.8 (1.3–2.7)**	0.52		**0.003 Male**	0.17 Male
Platelet count	0.17			0.40		0.41	
Platelet count ≥1000 × 10^9^/l	0.61			**0.01**	**0.05** **2.3 (1.0–5.2)**	0.08	0.29
Leukocyte count	**<0.0001**	**<0.0001**		0.85	0.25	0.72	
Leukocyte count >11 × 10^9^/l	**<0.0001**	**<0.0001**	**0.01** **1.6 (1.2–2.1)**	0.89	0.19	0.23	
Absolute neutrophil count ≥8 × 10^9^/l *N* evaluable = 653	**<0.0001**	**<0.0001**	**0.001** **2.4 (1.6–3.4)**	0.40		**0.04**	**0.01** **2.3 (1.2–4.3)**
Absolute lymphocyte count <1.7 × 10^9^/l *N* evaluable = 650	**0.01**	0.08	**0.02** **1.5 (1.1–2.1)**	0.16		0.99	
Driver mutation	**0.01** ***JAK2*** ***MPL***	0.76		0.96		**0.003** ***MPL***	**0.003** ***MPL*** **3.9 (1.8–8.4)**
Abnormal karyotype	**0.005**	**0.05**		**0.04**	**0.04** **3.1 (1.1–9.3)**	0.83	
Presence of cardiovascular risk factor*	**<0.0001**	**0.01**	**0.0001** **1.8 (1.3–2.5)**	0.16		0.39	
Hypertension *N* evaluable = 943	**<0.0001**	**0.02**	**0.01** **1.7 (1.1–2.6)**				
Arterial thrombosis at or prior to diagnosis	**0.0001**	**0.049**	**0.01** **1.7 (1.1–2.7)**	0.27		0.10	
Venous thrombosis at or prior to diagnosis	0.27			0.77		0.44	
Major hemorrhage at or prior to diagnosis	0.55			0.39		0.89	
Arterial thrombosis after diagnosis	**0.003**	0.15		0.66		0.98	
Venous thrombosis after diagnosis	0.96			0.08		0.53	
Major hemorrhage after diagnosis	**0.04**	0.61		0.18		0.96	

*Cardiovascular risk factor includes the presence of diabetes mellitus, hypertension, or smoking. Bold value boxes represent variables of significance.

**Fig. 2:**
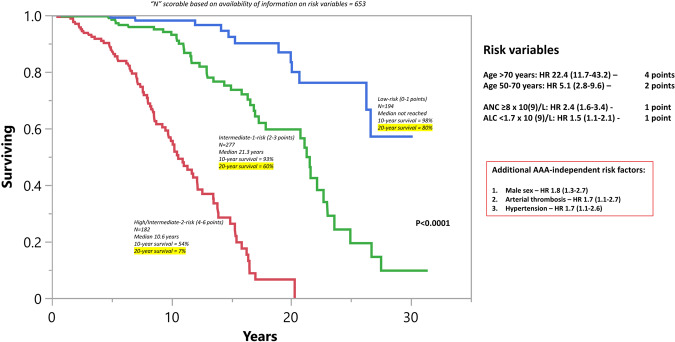
Triple A risk model in essential thrombocythemia.

Subsequently, a three-tiered “triple A” risk stratification model was applied in 653 informative patients with HR-weighted scoring, allocating four adverse points for age >70 years, two adverse points for age 50–70 years, one adverse point each for ANC ≥ 8 × 10^9^/L, and, ALC < 1.7 g/dl: low (0–1 point; *n* = 194), intermediate-1 (2–3 points; *n* = 277), and high/intermediate-2 (4–6 points; *n* = 182), with respective median survival (20-year rate) of not reached (80%), 21.3 years (60%), and 10.6 (7%) years (*p* < 0.0001) (Fig. 2).

#### Blastic transformation and fibrotic progression

**Fig. 3:**
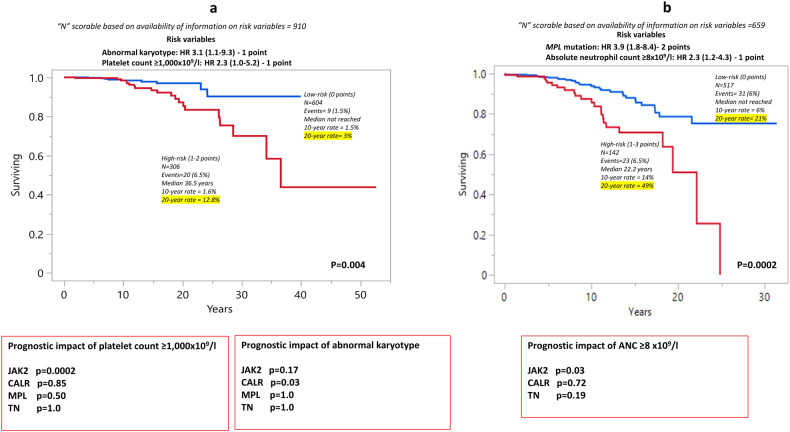
Blastic transformation and fibrotic progression in essential thrombocythemia. **a** Blastic transformation risk model in essential thrombocythemia. **b** Fibrotic progression risk model in essential thrombocythemia.

At the time of last follow-up in August 2023, blastic transformations were reported in 33 patients (3%), with overall incidence, 10-year and 20-year rates of 3%, 1.5% and 7.6%, respectively; 3%/1.7%/8% for *JAK2*, 3%/0.9%/0.9% for type 1/type 1-like *CALR*, 6%/3%/6% for type 2/type 2-like *CALR*, 3%/0%/0.2% with *MPL* and 0%/0%/0% with TN. There was no significant difference in LFS among the driver mutational categories (*p* = 0.29) (Fig. 1b). On the other hand, LFS was significantly worse in patients with ExT; platelet count ≥1000 × 10^9^/l (*p* = 0.05; HR 2.3, 95% CI 1.0–5.2) and abnormal karyotype (*p* = 0.03; HR 3.1, 95% CI 1.1–9.3) (Table 3). Based on the aforementioned findings, a two-tiered blastic transformation risk model was developed in 910 informative cases, allocating one adverse point each for platelet count ≥1000 × 10^9^/l and abnormal karyotype: low (0 points; *n* = 604), and high (1–2 points; *n* = 306), with respective median LFS (20-year rate) of not reached (3%), and 36.5 years (12.8%) (*p* = 0.004) (Fig. 3a). It is to be noted none of the TN patients succumbed to leukemic transformation and the latter’s association with ExT was limited to *JAK2*-mutated cases (*p* = 0.0002). On the other hand, abnormal karyotype was associated with inferior LFS in *CALR*-mutated cases (*p* = 0.03).

126 patients (13%) experienced transformation to myelofibrosis with 10-year and 20-year incidence rates of 9% and 30%, respectively. Overall incidence/10-year/20-year figures for fibrotic transformation were 10%/8%/26% for *JAK2-*mutated, 20%/13%/36% type 1/type 1-like *CALR-*mutated, 15%/5%/39% type 2/type 2-like *CALR*-mutated, 27%/35%/63% *MPL-*mutated and 10%/5%/19% TN cases (*p* < 0.01); the difference was significant for *MPL* vs *JAK2 (p* < 0.01, HR 4.0, 95% CI 1.9–8.4*), MPL* vs type 1/type 1-like and type 2/type 2-like *CALR* (*p* = 0.03 and 0.01, respectively, HR 2.4, 95% CI 1.1–5.2 and HR 3.7, 95% CI 1.6–8.7), type 1/type 1-like *CALR* vs *JAK2* (*p* = 0.02, HR 1.7, 95% CI 1.1–2.6), and type 1/type 1-like *CALR* vs TN (*p* = 0.03, HR 2.4, 95% CI 1.1–5.3) (Fig. 1c). Univariate analysis also identified age, male gender and ANC ≥ 8 × 10^9^/L as risk factors for fibrotic progression (*p* = 0.04/0.003/0.04). Multivariable analysis that included factors that were significant in univariate analysis confirmed the independent prognostic relevance of *MPL* mutations (*p* < 0.01; HR 3.9, 95% CI 1.8–8.4), and ANC ≥ 8 × 10^9^/L (*p* = 0.01; HR 2.3, 95% CI 1.2–4.3) for MFFS (Table 3). An HR-based risk model incorporating *MPL* mutation (2 points) and ANC ≥ 8 × 10^9^/L (1 point) delineated patients with median MFFS ranging from not reached to 22.2 years, and 20-year myelofibrosis incidence from 12% to 49%, in low (0 points) and high-risk (1–3 points) groups, respectively (Fig. 3b). Furthermore, the prognostic impact of ANC ≥ 8 × 10^9^/L was limited to *JAK2*-mutated cases (*p* = 0.03).

#### Arterial and venous thrombosis

**Fig. 4:**
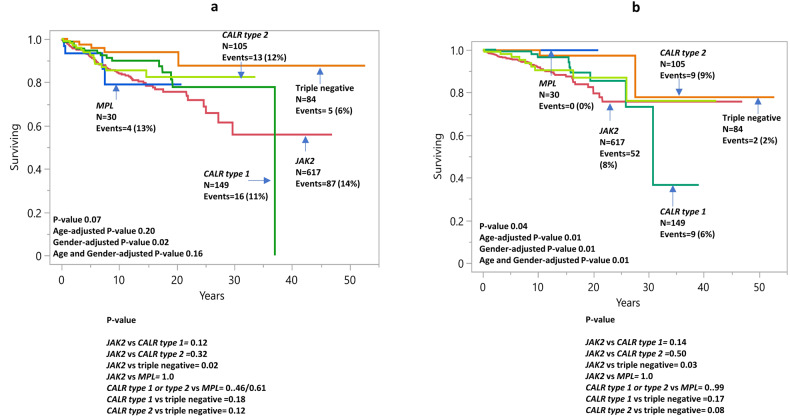
Arterial and venous thrombosis in essential thrombocythemia. **a** Arterial Thrombosis-free survival in 985 patients with essential thrombocythemia stratified by driver mutation. **b** Venous thrombosis-free survival in 985 patients with essential thrombocythemia stratified by driver mutation.

At a median follow-up time of 8.5 years (range, 0.01–52.7), major thrombosis after diagnosis was documented in 162 (16%) of patients including 127 (13%) arterial and 70 (7%) venous events. Incidence rates of major arterial/venous thrombosis for *JAK2*, type 1/type 1-like *CALR*, type 2/type 2-like *CALR, MPL*-mutated and TN cases were 14%/8%, 11%/6%, 12%/9%, 13%/0%, and 6%/2%, respectively. Figure 4a. illustrates arterial TFS stratified by driver mutations and discloses higher rates in *JAK-*mutated cases when compared with TN or type 1/type 1-like *CALR-*mutated (*p* = 0.02 and 0.12, respectively, HR 3.0, 95% CI 1.2–7.5 and HR 1.5, 95% CI 0.9–2.6). Arterial thrombosis rate was found to be similar in *JAK2* and *MPL*-mutated cases (*p* = 1.0); on the other hand, a non-significantly higher rate was observed in type 1/type 1-like *CALR* and type 2/type 2-like *CALR*-mutated patients in comparison with TN; *p* = 0.18 and *p* = 0.12, respectively, HR 1.9, 95% CI 0.7–5.4 and HR 2.3, 95% CI 0.8–6.4 (Table 4). Findings were unchanged when analysis accounted for age and gender differences among the driver mutation categories (Table 4). In addition, univariate analysis identified age ≥60 years, male gender, leukocyte count >11 × 10^9^/l, hypertension, and arterial thrombosis history as predictors of inferior arterial TFS. Multivariable analysis confirmed age ≥60 years (*p* = 0.001), male gender (*p* = 0.01), *JAK2* mutational status (*p* = 0.01), hypertension (*p* = 0.01), and arterial thrombosis history (*p* = 0.02) as independent predictors of future arterial thrombotic events (Table 2).

**Table 4:** Univariate and multivariable analysis of risk factors for vascular events (arterial/venous thrombosis and hemorrhage) after diagnosis among 985 patients with essential thrombocythemia (ET), fully annotated for driver mutations with *CALR* mutation type.

Follow-up in years, median (range): 8.5 (0.01–52.7)	Arterial thrombosis-free survival			Venous thrombosis-free survival			Hemorrhage-free survival		
	Events; *n* (%): 125 (13)			Events*; n* (%): 72 (7)			Events; *n* (%): 107/968 (11)		
Driver mutations	Univariate *P* value HR (95% CI)	Age-adjusted *P* value HR (95% CI)	Gender-adjusted *P* value HR (95% CI)	Univariate *P* value HR (95% CI)	Age-adjusted *P* value HR (95% CI)	Gender-adjusted *P* value HR (95% CI)	Univariate *P* value HR (95% CI)	Age-adjusted *P* value HR (95% CI)	Gender-adjusted *P* value HR (95% CI)
JAK2 vs type 1 CALR	0.12 1.5 (0.9–2.6)	0.37	**0.05** **1.7 (1.0–2.9)**	0.14 1.7 (0.8–3.5)	0.19 1.6 (0.8–3.3)	0.06 1.9 (0.9–4.1)	0.08 1.7 (0.9–3.2)	0.21	**0.05** **1.9 (1.0–3.5)**
JAK2 vs type 2 CALR	0.32	0.81	0.21	0.50	0.64	0.36	0.37	0.77	0.29
JAK2 vs MPL	0.99	0.67	0.88	0.99	0.99	0.99	0.12 0.51 (0.2–1.2)	0.25	0.15 0.54 (0.2–1.3)
JAK2 vs triple negative	**0.02** **3.0 (1.2–7.5)**	**0.04** **2.6 (1.03–6.3)**	**0.02** **3.0 (1.2–7.4)**	**0.03** **4.9 (1.2–20.2)**	**0.03** **4.6 (1.1–19.0)**	**0.03** **4.9 (1.2–20.2)**	0.12 1.9 (0.8–4.1)	0.25	0.12 1.8 (0.8–4.0)
Type 1 CALR vs Type 2 CALR	0.73	0.64	0.65	0.54	0.52	0.46	0.58	0.49	0.52
Type 1 CALR vs MPL	0.46	0.96	0.41	0.99	0.99	0.99	**0.02** **0.29 (0.1–0.8)**	0.08 0.4 (0.2–1.1)	**0.01** **0.3 (0.1–0.8)**
Type 2 CALR vs MPL	0.61	0.80	0.61	0.99	0.99	0.99	0.06 0.4 (0.1–1.0)	0.26	0.06 0.4 (0.1–1.1)
Type 1 CALR vs triple negative	0.18 1.9 (0.7–5.4)	0.18 1.9 (0.7–5.5)	0.28	0.17 2.9 (0.6–13.5)	0.18 2.9 (0.6–13.3)	0.25	0.86	0.88	0.96
Type 2 CALR vs triple negative	0.12 2.3 (0.81–6.4)	0.10 2.4 (0.84–6.7)	0.17 2..1 (0.7–5.9)	0.08 3.9 (0.8–17.9)	0.08 3.9 (0.8–18.1)	0.11 3.5 (0.8–16.3)	0.52	0.46	0.61
MPL vs triple negative	0.10 3.0 (0.81–11.2)	0.28	0.13 2.8 (0.7–10.4)	0.99	0.99	0.99	**0.02** **3.6 (1.2–10.9)**	0.09 2.6 (0.9–7.6)	**0.03** **3.4 (1.1–10.2)**

Bold value boxes represent variables of significance.

A separate analysis of venous TFS also disclosed higher risk in *JAK2* and *MPL-*mutated patients, when compared with TN and type 1/type 1-like *CALR-*mutated (*p* = 0.03 and 0.14, respectively, HR 4.9, 95% CI 1.2–20.3 and HR 1.7, 95% CI 0.8–3.5) (Fig. 3b); additionally, we observed a trend for higher risk of venous thrombosis among type 1/type 1-like *CALR* and type 2/type 2-like *CALR-*mutated when compared with TN cases; *p* = 0.17 and *p* = 0.08, respectively, HR 2.9, 95% CI 0.6–13.3 and HR 3.9, 95% CI 0.8–17.9 (Table 4). Multivariable analysis inclusive of age, gender, venous thrombosis history, and driver mutation category, identified male gender, and venous thrombosis history as independent predictors of venous thrombosis (*p* = 0.02/< 0.01) (Table 2).

Aspirin therapy appeared to mitigate both arterial and venous thrombosis with arterial and venous thrombosis rates of 5% vs 16% and 3% vs 9% in patients receiving or not receiving aspirin (*p* < 0.01 and *p* = 0.02, respectively, HR 0.4, 95% CI 0.2–0.8 and HR 0.4, 95% CI 0.2–0.9). Additional analyses revealed that the apparent differences in arterial and venous thrombosis observed among type 1/type 1-like *CALR-*mutated and TN cases, were fully accounted for by aspirin use (*p* value adjusted for aspirin use = 0.40/0.31). Cytoreductive therapy, on the other hand, did not appear to have a clear beneficial impact on neither arterial (*p* = 0.08) nor venous thrombosis (*p* = 0.19).

#### Major hemorrhage

A total of 107 major hemorrhagic events were recorded in 983 patients (11%); 33 of 108 (31%) evaluable patients (including 7 of 22 (32%) with major hemorrhage) had laboratory evidence of acquired von Willebrand syndrome. Incidence rates of major hemorrhage were 12%, 8%, 10%, 20%, and 9%, for *JAK2*, type 1/type 1-like *CALR*, type 2/type 2-like *CALR, MPL*-mutated and TN cases, respectively, with higher rates among *MPL*-mutated cases compared to type 1/type 1-like *CALR*, type 2/type 2-like *CALR* and TN (*p* = 0.02/0.06/0.02) (Table 4). Additionally, a higher incidence of major hemorrhage was also observed in *JAK2*-mutated patients compared to type 1/type 1-like *CALR-*mutated (*p* = 0.08, HR 1.7, 95% CI 0.9–3.2). Furthermore, on univariate analysis, age ≥60 years (*p* < 0.01), leukocyte count >11 × 10^9^/l (*p* < 0.01), presence of cardiovascular risk factors (*p* < 0.01), and history of major hemorrhage (*p* = 0.01) predicted future hemorrhage, while aspirin use was of borderline significance (*p* = 0.10). On multivariable analysis, age ≥60 years (*p* < 0.01), leukocyte count >11 × 10^9^/l (*p* < 0.01), and presence of cardiovascular risk factors (*p* = 0.05) remained significant predictors of major hemorrhage after diagnosis.

## Discussion

The current study constitutes the largest single-center series of ET patients who are fully annotated for driver mutations and includes mature survival data and detailed analysis of prognostic factors for overall, leukemia-free, myelofibrosis-free, and thrombosis-free survival, with the latter stratified into arterial vs. venous events. Our study provides baseline clinical and laboratory data and confirms previously recognized differences in age and gender distribution as well as hemoglobin, leukocyte, and platelet levels, among specific driver mutation categories; *CALR-*mutated and TN patients were younger at diagnosis (median age 52 and 50 years, respectively), compared to *JAK2* or *MPL*-mutated cases (median age 71 and 66 years, respectively); *JAK2* and TN patients were predominantly female, compared to *CALR* and *MPL*-mutated cases [19]. Noteworthy laboratory associations included higher hemoglobin and leukocyte count with JAK2 mutation and higher platelet count with *CALR* mutation (type-2 more than type-1) and TN [19]. At the time of diagnosis, approximately 22% of patients displayed history of major arterial (14%) or venous (10%) thrombosis, 8% major hemorrhage, and 29%, microvascular symptoms; incidences of major thrombosis and hemorrhage were lower in *CALR*-mutated cases [20].

A major strength of the current study was the availability of long-term follow-up data, which enabled accurate estimation of survival and disease transformation rates; median overall survival was 20.6 years, with 10-year/20-year leukemic transformation and fibrotic progression rates of 1.5%/7.6% and 8%/26%, respectively. As previously noted [9], *JAK2*/*CALR*/*MPL*/TN mutational status did not appear to impact overall or leukemia-free survival in our current ET patient cohort while *MPL* mutation was associated with a significantly higher rate of progression to myelofibrosis, as per previous reports [21, 22]. Prominent risk factors for survival in the current ET patient cohort included older age, ANC ≥ 8 × 10^9^/L, ALC < 1.7 × 10^9^/L, male gender, hypertension, and arterial thrombosis history. These observations are in line with those previously communicated [10, 23]. Application of the recently introduced AAA survival model in ET [10], to the current patient cohort resulted in median survival estimates not reached for low (10-/20-year survival rate 98%/80%), 21.3 years for intermediate-1 (10-/20-year survival rate 93%/60%), and 10.6 years for high/intermediate-2 (10-/20-year survival rate 54%/7%) risk patients (Fig. 1).

Additional observations from the current study are highlighted by (i) the extremely low incidence of leukemic transformation in the absence of abnormal karyotype and ExT (10-/20-year rate of 1.5%/3%), and (ii) the relatively high rate of fibrotic progression in *MPL*-mutated patients or those with ANC ≥ 8 × 10(9)/L. It is to be recalled that we have previously reported an association between ExT and inferior overall and leukemia-free survival in young (age <40 years) ET patients [24]. However, the association between ExT and leukemic progression in the current study appeared to be limited to patients with *JAK2* mutation; this is a noteworthy observation, given that ExT is typically associated with *CALR* (type-2 more than type-1) and TN mutational status. Interestingly, none of our 84 TN patients with ET experienced leukemic progression. These findings require external validation. The prognostic impact of ANC ≥ 8 × 10^9^/L on MFFS, has not been previously described, and was limited to *JAK*-mutated cases. Furthermore, in line with prior reports, type 1/type 1-like *CALR* mutated compared to *JAK2*-mutated, and TN cases were noted to have a significantly higher risk of fibrotic progression [22]. The current study did not include information on other mutations that have previously been shown to adversely affect survival, including *SF3B1*, *SRSF2 U2AF1 TP53* mutations [12].

The current study also confirms the higher rates of arterial and venous thrombosis displayed by *JAK2*/*MPL*-mutated patients with ET, compared with type 1/type 1-like *CALR-*mutated and TN, and that this effect was not accounted for by other independent risk factors for thrombosis including age, gender, thrombosis history or cardiovascular risk factors [20]. Moreover, a non-significantly higher risk of thrombosis was also observed in *CALR*-mutated patients in comparison with TN cases; which was no longer apparent after accounting for aspirin use. Together, our findings not only confirm differential thrombosis risk according to driver mutation, but also identify TN patients to have a lower risk of thrombosis, akin to those with *CALR* mutation. Our observations corroborate the salutary effects of aspirin with respect to both arterial and venous thrombosis, and also suggest possible benefits from aspirin prophylaxis in “*very low risk” CALR*-mutated patients.

From a practical standpoint, the prognostic information discussed in the current report, regarding survival is not necessarily actionable, since drug therapy in ET has not been shown to be disease-modifying. However, identification of prognostic factors for survival and disease progression is useful for purposes of patient counseling and disease monitoring. On the other hand, risk factor assessment for thrombosis is critical for both primary and secondary prevention measures. In this regard, the observations from the current study underscore the therapeutic value of aspirin for the prevention of both arterial and venous thrombosis. Controlled studies are always preferred over retrospective studies for accurate determination of the optimal therapeutic approach to ameliorate the risk associated with thrombosis and disease progression in ET. In the meantime, we hope the information contained in the current document serves as a complimentary resource for patients and physicians and provides context for the design and interpretation of future clinical trials [12, 25–29].

### Supplementary information

Supplemental Table 1
