# Supplementary material for: One thousand patients with essential thrombocythemia: the Mayo Clinic experience
Source: Blood Cancer J. 2024 Jan 18;14(1):11. doi: 10.1038/s41408-023-00972-x (PMC10796913; doi:10.1038/s41408-023-00972-x)
Supplement: Supplementary file 1 — Supplemental Table 1 [file 41408_2023_972_MOESM1_ESM.docx]

**Supplemental Table 1: Presenting clinical and laboratory characteristics of 871 patients with essential thrombocythemia (ET), fully annotated for driver mutations, stratified by *JAK2 vs* Type 1/type 1-like vs Type 2/type 2-like *CALR* mutation**

*n*=254 of 269 patients were informative for type of *CALR* mutation

| **Variables** | **All patients**  ***n*=871** | ***JAK2* mutated**  ***n* =617 (71%)** | ***Type 1/type 1- like***  ***CALR*  mutated**  ***n*=149 (17%)** | ***Type 2/type 2- like CALR* mutated**  ***n*=105 (12%)** | ***P-value***  ***JAK2 vs Type 1/type 1- like***  ***CALR*** | ***P-value***  ***JAK2 vs Type 2/type-2 like***  ***CALR*** | ***P-value***  ***Type 1/type 1- like vs Type 2/type 2- like CALR*** |
| --- | --- | --- | --- | --- | --- | --- | --- |
| Age in years, median (range)   - Age ≥ 60 years, *n* (%) | 59 (18-90)  394 (45) | 71 (18-90)  305 (49) | 53 (18-83)  52 (35) | 51 (18-85)  37 (35) | **<0.0001**  **0.001** | **0.004**  **0.01** | 0.90  0.96 |
| Female gender, *n* (%) | 556 (64) | 427 (69) | 75 (50) | 54 (51) | **<0.0001** | **0.001** | 0.86 |
| Hemoglobin g/dl, median (range) | 13.9 (10-17.2) | 14.0 (10-17.2) | 13.7 (11.4-16.4) | 13.4 (10.5-16.3) | 0.06 | **0.001** | 0.15 |
| Leukocyte count, 10^9^/l, median (range)  - Leukocyte count > 11 x 10^9^/l, *n* (%) | 8.6 (3.5-23)  179 (21) | 8.9 (3.5-23)  147 (24) | 8.1 (4-17.7)  18 (12) | 7.9 (3.5-18.6)  14 (13) | **<0.0001**  **0.001** | **0.003**  **0.01** | 0.72  0.77 |
| Platelet count, 10^9^/l, median (range)  - Platelet count ≥ 1000 x 10^9^/l, *n* (%)  - Platelet count ≥ 1500 x 10^9^/l, *n* (%) | 753 (450-3460)  221 (25)  38 (4) | 705 (450-2466)  105 (17)  16 (3) | 890 (454-3460)  58 (39)  6 (4) | 1044 (467-2851)  58 (55)  16 (15) | **<0.0001**  **<0.0001**  0.37 | **<0.0001**  **<0.0001**  **<0.0001** | **0.001**  **0.01**  **0.002** |
| Cardiovascular risk factors, *n* (%)  -Diabetes mellitus  -Hypertension  -Smoking | 440/822 (54)  69/821 (8)  355/822 (43)  158/820 (20) | 334/588 (57)  49/588 (8)  271/588 (46)  124/587 (21) | 63/142 (44)  8/142 (6)  51/142 (36)  17/143 (12) | 43/92 (47)  12/91 (13)  33/92 (36)  17/90 (19) | **0.01**  0.26  **0.03**  **0.01** | 0.07  0.15  0.06  0.62 | 0.72  **0.05**  0.99  0.14 |
| Palpable splenomegaly, *n* (%) | 106/862 (12) | 77/609 (13) | 13/149 (9) | 16/104 (15) | 0.17 | 0.45 | 0.10 |
| Abnormal karyotype, *n* (%) | 49/763 (6) | 40/546 (7) | 5/126 (4) | 4/91 (4) | 0.15 | 0.28 | 0.88 |
| Major thrombosis at or prior to diagnosis, *n* (%)   - Arterial thrombosis^*^ - Venous thrombosis^#^ | 191 (22)  116 (13)  90 (10) | 164 (27)  98 (16)  80 (13) | 18 (12)  10 (7)  8 (5) | 9 (9)  8 (7)  2 (2) | **<0.0001**  **0.002**  **0.004** | **<0.0001**  **0.02**  **<0.0001** | 0.37  0.78  0.14 |
| Major hemorrhage at or prior to diagnosis^€^, *n* (%) | 60/856 (7) | 47/609 (8) | 6/148 (4) | 7/99 (7) | 0.10 | 0.82 | 0.30 |
| Microvascular symptoms^∞^, *n* (%) | 250/835 (30) | 180/596 (30) | 41/145 (28) | 29/94 (31) | 0.65 | 0.90 | 0.67 |
| Revised IPSET-thrombosis^¥^, *n* (%)   - Very Low - Low - Intermediate - High | 43 (5)  202 (23)  157 (18)  469 (54) | 0 (0)  79 (13)  94 (15)  444 (72) | 26 (17)  71 (48)  35 (24)  17 (11) | 17 (16)  52 (50)  28 (27)  8 (8) | - | - | 0.73 |
| IPSET-survival^µ^, *n* (%)   - Low - Intermediate - High | 323 (37)  379 (44)  169 (19) | 187 (30)  290 (47)  140 (23) | 80 (54)  51 (34)  18 (12) | 56 (53)  38 (36)  11 (10) | **<0.0001** | **<0.0001** | 0.90 |
| Treatment instituted at diagnosis, *n* (%)   - Aspirin - Cytoreductive therapy^$^ - Systemic anticoagulation | 644/793 (81)  489/798 (61)  150/741 (20) | 482/575 (84)  340/574 (59)  119/527 (23) | 104/133 (78)  90/136 (66)  16/128 (13) | 58/85 (68)  59/88 (67)  15/86 (17) | 0.13  0.13  **0.01** | **0.001**  0.16  0.27 | 0.10  0.89  0.32 |

*Major arterial thrombosis includes myocardial infarction, angina, cerebrovascular accidents, transient ischemic attack, peripheral arterial thrombosis, aortic thrombosis, mesenteric artery thrombosis, central retinal thrombosis.

# Major venous thrombosis includes deep venous thrombosis, pulmonary embolism, portal/splenic/mesenteric/hepatic vein thrombosis, cerebral sinus thrombosis

€ Major hemorrhage includes bleeding events that require red cell transfusion support, resulted in ≥2 g/dl decline in hemoglobin or involved critical organs

∞ Microvascular symptoms include headaches, paresthesia, erythromelalgia.

¥ International prognostic score for thrombosis in ET (IPSET-thrombosis).

µ International prognostic score for survival in ET (IPSET-survival).

$ cytoreductive therapies included hydroxyurea, anagrelide, interferon, busulphan
